# Algorithm-agnostic significance testing in supervised learning with multimodal data

**DOI:** 10.1093/bib/bbae475

**Published:** 2024-09-25

**Authors:** Lucas Kook, Anton Rask Lundborg

**Affiliations:** Institute for Statistics and Mathematics, Vienna University of Economics and Business, Welthandelsplatz 1, AT-1020 Vienna, Austria; Department of Mathematical Sciences, University of Copenhagen, Universitetsparken 5, DK-2100 Copenhagen, Denmark

**Keywords:** Conditional independence, Generalised Covariance Measure, multimodal data, Projected Covariance Measure, significance testin

## Abstract

**Motivation:**

Valid statistical inference is crucial for decision-making but difficult to obtain in supervised learning with multimodal data, e.g. combinations of clinical features, genomic data, and medical images. Multimodal data often warrants the use of black-box algorithms, for instance, random forests or neural networks, which impede the use of traditional variable significance tests.

**Results:**

We address this problem by proposing the use of COvariance MEasure Tests (COMETs), which are calibrated and powerful tests that can be combined with any sufficiently predictive supervised learning algorithm. We apply COMETs to several high-dimensional, multimodal data sets to illustrate (i) variable significance testing for finding relevant mutations modulating drug-activity, (ii) modality selection for predicting survival in liver cancer patients with multiomics data, and (iii) modality selection with clinical features and medical imaging data. In all applications, COMETs yield results consistent with domain knowledge without requiring data-driven pre-processing, which may invalidate type I error control. These novel applications with high-dimensional multimodal data corroborate prior results on the power and robustness of COMETs for significance testing.

**Availability and implementation:**

COMETs are implemented in the cometsR package available on CRAN and pycometsPython library available on GitHub. Source code for reproducing all results is available at https://github.com/LucasKook/comets. All data sets used in this work are openly available.

## Introduction

A fundamental challenge of modern bioinformatics is dealing with the increasingly multimodal nature of data [1–3]. The task of *supervised learning*, that is, the problem of predicting a response variable $Y$ from features $X$, has received considerable attention in recent years resulting in a plethora of algorithms for a wide range of settings that permit prediction using several data modalities simultaneously [4]. With the advent of deep learning, even non-tabular data modalities, such as text or image data, can be included without requiring manual feature engineering [5]. Methods such as these are highly regularized (if trained correctly) which minimizes the statistical price of adding too many irrelevant variables. However, continuing to collect features or modalities that do not contribute to the predictiveness of a model still has an economic cost and, perhaps more importantly, it is of scientific interest to determine whether a particular feature or modality $X$ adds predictive power in the presence of additional features or modalities $Z$ [6].

The problem of determining which features or modalities are significantly associated with the response is usually addressed by means of *conditional independence testing*. The response $Y$ is independent of the modality $X$ given further modalities $Z$ if the probability that $Y$ takes any particular value knowing both $X$ and $Z$ is the same as the probability knowing just $Z$. In particular, $X$ does not help in predicting $Y$ if $Z$ is taken into account already (see Background on conditional independence for a more precise definition).

Traditional variable significance tests start by posing a parametric relationship between the response $Y$ and features $X$ and $Z$, for instance, the Wald test in a generalized linear model. When $X$ or $Z$ are complicated data modalities, it is seldom possible to write down a realistic model for their relationship with $Y$; thus a different approach is required. Furthermore, even when models can be explicitly parametrized, it is not clear that the resulting tests remain valid when the model is not specified correctly [7].

More recently, kernel-based conditional independence tests have been proposed which use a characterization of conditional independence by means of kernel embeddings to construct tests [8, 9]. However, these tests are difficult to calibrate in practice and rely intimately on kernel ridge regression. Several alternative algorithm-agnostic tests have been developed under the so-called ‘Model-X’ assumption where one supposes that a model is known (or at least estimable to high precision) for the full distribution of $X$, given $Z$ [10, 11]. Given the difficulty of learning conditional distributions such an assumption is rarely tenable. Algorithm-agnostic variable importance measures have also been developed with statistically optimal estimators [12, 13]. However, efficient estimation of an importance measure does not necessarily translate to an optimal test to distinguish between conditional dependence and independence [see, e.g. the introduction of 14].

**Figure 1:**
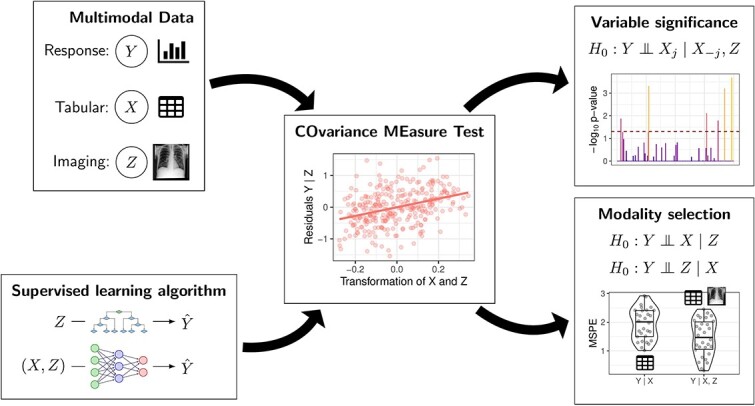
Overview of the proposed algorithm-agnostic significance testing framework for multimodal data using COMETs. **Variable significance**: differential gene expression can be assessed in presence of the potentially high-dimensional/non-tabular confounder $Z$. **Modality selection**: entire modalities can be subjected to significance testing, which lends itself to modality selection in multi-omics applications. MSPE: mean squared prediction error.

In this paper, we describe a family of significance tests referred to collectively as COvariance MEasure Tests (COMETs) that are algorithm-agnostic and valid (in the sense of controlling the probability of false positives) as long as the algorithms employed are sufficiently predictive. We will primarily focus on the Generalised Covariance Measure (GCM) test [15], which we think of as an ‘all-purpose’ test that should be well-behaved in most scenarios, and the more complicated Projected Covariance Measure (PCM) test, which is more flexible but may require a more careful choice of algorithms. Figure 1 gives an overview of the proposed algorithm-agnostic significance testing framework based on COMETs and the types of applications that are presented in this manuscript. The main contribution of this work is to illustrate the use of the GCM and PCM test in the context of multimodal, non-tabular data.

## Methods

In this section, we first provide some background on conditional independence. We then move on to describe the computation of the GCM and PCM tests in addition to the assumptions required for their validity. Finally, we describe the datasets that we will analyze in Results.

### Background on conditional independence

For a real-valued response $Y$ and features $X$ and $Z$, we say that $Y$ is *conditionally independent of $X$, given $Z$* and write $Y \perp \hspace{-5pt}\perp X \,| \, Z$ if 


(1) \begin{align*}& {\mathbb{E}}[h(Y) \,|\, X, Z] = {\mathbb{E}}[h(Y) \,|\, Z] \quad \text{for all functions}\ {h}.\end{align*}

That is, for any transformation $h$ of $Y$, the best predictor (in a mean-squared error sense) of $h(Y)$ using both $X$ and $Z$ is equal to the best predictor using just $Z$.(An alternative characterization, when $Y$ has a conditional density given $X$ and $Z$ denoted by $f_{Y|X,Z}(y | x, z)$, is given by: $f_{Y|X,Z}(y|x,z) = f_{Y|Z}(y|z)$ if and only if $Y$ is independent of $X$, given $Z$.).

A helpful starting point for the construction of a conditional independence test is to consider the product of a population residual from a $Y$ on $Z$ regression $\varepsilon := Y- {\mathbb{E}}[Y \,|\, Z]$ and, for now considering a one-dimensional $X$, from an $X$ on $Z$ regression $\xi := X - {\mathbb{E}}[X \,|\, Z]$. As these are population residuals, $Z$ is no longer helpful in predicting their values, so ${\mathbb{E}}[\varepsilon \,|\, Z] = {\mathbb{E}}[\varepsilon ] = 0$ and similarly ${\mathbb{E}}[\xi \,|\, Z] =0$. When $Y \perp \hspace{-5pt}\perp X \,|\, Z$, we can say more: the product of the residuals is also mean zero since 


(2) \begin{align*} {\mathbb{E}}[\varepsilon \xi ] = {\mathbb{E}}[{\mathbb{E}}[\varepsilon \xi \,|\, X, Z]] = {\mathbb{E}}[{\mathbb{E}}[\varepsilon \,|\, X, Z] \xi ] = {\mathbb{E}}[{\mathbb{E}}[\varepsilon \,|\, Z] \xi ] = 0,\end{align*}

where the second equality uses that $\xi $ is perfectly predicted using $X$ and $Z$, the third equality uses (1) with $h(y) = y$ and the final equality uses ${\mathbb{E}}[\varepsilon \,|\, Z] = 0$. The GCM test is based on testing whether ${\mathbb{E}}[\varepsilon \xi ] = 0$ and we will describe the details of how to compute it in Covariance measure tests. For the GCM test to perform well, it is important to determine when we can expect ${\mathbb{E}}[\varepsilon \xi ]$ to be non-zero under conditional dependence. When $Y$ follows a partially linear model given $X$ and $Z$, that is, ${\mathbb{E}}[Y \,|\, X, Z] = \theta X + h(Z)$ for some function $h$, then ${\mathbb{E}}[\varepsilon \xi ] \neq 0$ exactly when $\theta \neq 0$ and the magnitude of ${\mathbb{E}}[\varepsilon \xi ]$ is proportional to $\theta $. This includes as a special case the linear model for $Y$, given $X$ and $Z$. There is a natural generalization of (2) to the case where $X$ is a $d$-dimensional vector, where the equation is interpreted component-wise in $X$. Although the GCM is also defined in these settings, computing the test involves many regressions when $X$ is high-dimensional, which can be impractical (see Comparison of the GCM and PCM tests).

**Figure 2:**
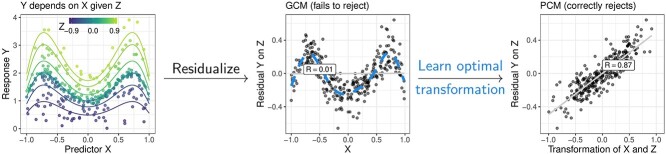
Illustration of the GCM and PCM test under the alternative that $Y \in{\mathbb{R}}$ is not conditionally independent of $X \in{\mathbb{R}}$, given $Z \in{\mathbb{R}}$, where $Y := f(X) g(Z) + \varepsilon $ and $f(x):= 1 + \sin (3x^{2})$, $g(z):= 1 + z^{3}$. The GCM test first computes the residual for the regression of $Y$ on $Z$, which shows no correlation with $X$. Thus, the GCM fails to reject (correlation coefficient $R = 0.01$). The PCM, in addition, learns the optimal transformation of $X$ (depending on $Z$) to test conditional mean independence of $Y$ and $X$, given $Z$. Thus, in this example, the PCM test correctly rejects ($R = 0.87$). Although the residuals in the second panel are clearly not independent, it is not valid to conclude conditional dependence from rejecting an independence test here [see 15, Example 1].

Unfortunately, it is not difficult to come up with examples where $Y \not \perp \hspace{-5pt}\perp X \,|\, Z$ but ${\mathbb{E}}[\varepsilon \xi ] = 0$. For instance, if $X$ and $Z$ are independent and standard normally distributed and ${\mathbb{E}}[Y \,|\, X, Z] = X^{2}$, then ${\mathbb{E}}[Y \,|\, Z] = 1$ (since $Z$ carries no information about $X$ so the best predictor is just the mean of $Y$); hence, 


\begin{align*} & {\mathbb{E}}[\varepsilon\xi] = {\mathbb{E}}[(X^{2} - 1) X] = 0, \end{align*}

using that ${\mathbb{E}}[X] = {\mathbb{E}}[X^{3}] = 0$ for a standard normal variable. A more elaborate example is given in Fig. 2 (left and middle panel) and even more examples exist when $X$ and $Z$ are dependent (see [14], Section 6 and [16], Section 3.1.2). We now describe a test that can detect such dependencies.

A more ambitious target is to detect whenever an arbitrary (e.g. non-tabular) $X$ is helpful for the prediction of $Y$ in the presence of $Z$ measured in terms of mean-squared error. To achieve this goal, we can use the fact, derived in the same way as (2), that 


\begin{align*} & {\mathbb{E}}[\varepsilon f(X, Z)] = 0 \quad \text{for all functions}\ {f}, \end{align*}

whenever $Y \perp \hspace{-5pt}\perp X \,|\, Z$. The GCM targets the quantity involving the function $f(X, Z) = X$. However, by instead using $f(X, Z) = {\mathbb{E}}[Y \,|\, X, Z] - {\mathbb{E}}[Y \,|\, Z]$ (which depends on the joint distribution of $X$ and $Z$), we obtain that 


(3) \begin{align*}& {\mathbb{E}}[\varepsilon f(X, Z)] = {\mathbb{E}}[({\mathbb{E}}[Y \,|\, Z] - {\mathbb{E}}[Y \,|\, X, Z])^{2}] =: \tau.\end{align*}

This quantity is strictly greater than $0$ if and only if $X$ is helpful for the prediction of $Y$ in the presence of $Z$. The PCM test is based on testing whether $\tau = 0$ and we will describe the details of how to compute it in Covariance measure tests. In fact, the PCM is based on an alternative $f$, given by $f(X, Z) = ({\mathbb{E}}[Y \,|\, X, Z] - {\mathbb{E}}[Y \,|\, Z])/\operatorname{Var}(Y \,|\, X, Z)$ that turns out to result in a more powerful test [see Fig. 2 and 14, Section 1.1]. An added benefit of tests targeting $\tau $ is that no regressions are needed with $X$ as the response, which can vastly reduce the computational burden when compared to tests that target ${\mathbb{E}}[\varepsilon \xi ]$.

The targets mentioned above rely intimately on population quantities that are unknown and hence need to be estimated when computing tests in practice. To ensure that the estimation errors do not interfere with the performance of the tests, we need to be able to learn the functions to a sufficient degree of accuracy. These requirements put restrictions on when the GCM and PCM are valid tests but such restrictions are not unique to these tests. In fact, unless $Z$ is discrete, it is impossible to construct an assumption-free conditional independence test that simultaneously controls the probability of false rejections and is able to detect dependence [15, 17]. This result implies that additional assumptions need to be imposed to ensure the feasibility of testing for conditional independence.

### Covariance measure tests

We now describe the specifics of computing the GCM and the PCM. For the remainder of this section, we assume that we have a dataset consisting of $n$ independent observations of a real-valued response $Y$ and some additional features or modalities $X$ and $Z$.

#### GCM test

The GCM test is based on (2) but to compute the test in practice, we need to form an empirical version of the equation. For simplicity, we consider, for now, $X \in \mathbb{R}$. Let $\widehat{\xi }_{i}$ denote the residual for the $i$th observation from regressing $X$ on $Z$ and similarly $\widehat{\varepsilon }_{i}$ from regressing $Y$ on $Z$. We now test $H_{0}: {\mathbb{E}}[\varepsilon \xi ] = 0$ by comparing 


(4) \begin{align*}& T:= \frac{\left(\frac{1}{\sqrt{n}} \sum_{i=1}^{n} \widehat{\varepsilon}_{i} \widehat{\xi}_{i}\right)^{2}}{\frac{1}{n} \sum_{i=1}^{n} \widehat{\varepsilon}_{i}^{2}\widehat{\xi}_{i}^{2} - \left(\frac{1}{n} \sum_{i=1}^{n} \widehat{\varepsilon}_{i}\widehat{\xi}_{i}\right)^{2}}\end{align*}

to a $\chi ^{2}_{1}$ distribution. The term inside the square in the numerator is $\sqrt{n}$ times an estimate of (2) while the denominator standardizes the variance of the test statistic. The test statistic in (4) is approximately $\chi ^{2}_{1}$ for large enough sample sizes if the regression methods employed are sufficiently predictive and $Y \perp \hspace{-5pt}\perp X \,|\, Z$ [15, Theorem 6]. Note that the procedure above did not use anything special about $Z$ other than the existence of a regression method that can approximate the conditional expectations of $Y$ and $X$, given $Z$. The computations above naturally generalize to settings where $X \in \mathbb{R}^{d}$ and we summarize the general procedure in Algorithm 1. 


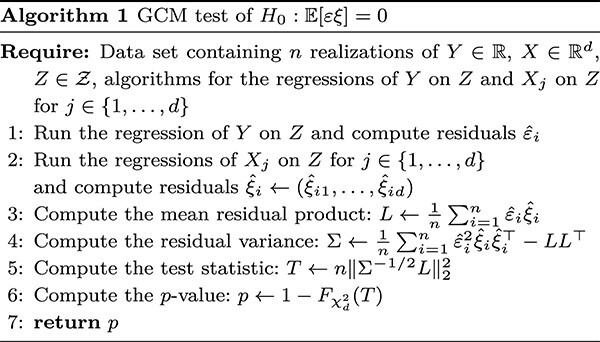


#### PCM test

The computation of the PCM test is more challenging than the computation of the GCM test since the PCM requires learning $f(X, Z) = ({\mathbb{E}}[Y \,|\, X, Z] - {\mathbb{E}}[Y \,|\, Z]) /\operatorname{Var}(Y \,|\, X, Z)$ to be able to estimate ${\mathbb{E}}[\varepsilon f(X, Z)]$. Furthermore, $f$ cannot be learned on the same observations that are used to compute the test statistic as this would potentially result in dependence between the residuals constituting the test statistic and thus in many false rejections when $Y \perp \hspace{-5pt}\perp X \,|\, Z$.

The first step when computing the test statistic of the PCM test is therefore to split the dataset in two halves $D_{1}$ and $D_{2}$ of equal size (for simplicity, we assume that we have $2n$ observations, so both $D_{1}$ and $D_{2}$ are of size $n$). On $D_{2}$, we compute an estimate $\widehat{f}$ of $f$ by first regressing $Y$ on $X$ and $Z$ yielding an estimate $\widehat{g}$ and regressing $Y$ on $Z$ yielding an estimate $\widehat{m}$. We then regress $(Y - \widehat{g}(X, Z))^{2}$ on $(X, Z)$ on $D_{2}$ yielding an estimate of $\operatorname{Var}(Y \,|\, X, Z)$, which we denote $\widehat{v}$. We now set $\widehat{f}(x, z):= (\widehat{g}(x, z) - \widehat{m}(z))/\widehat{v}(x, z)$ and, working on $D_{1}$, we regress $Y$ on $Z$ yielding a residual for the $i$th observation $\widehat{\varepsilon }_{i}$ and we regress $\widehat{f}(X, Z)$ on $Z$ yielding a residual $\widehat{\zeta }_{i}$. Finally, we compute 


(5) \begin{align*}& T:= \frac{\frac{1}{\sqrt{n}} \sum_{i=1}^{n} \widehat{\varepsilon}_{i}\widehat{\zeta}_{i}}{ \left(\frac{1}{n} \sum_{i=1}^{n} \widehat{\varepsilon}_{i}^{2}\widehat{\zeta}_{i}^{2} - \left(\frac{1}{n} \sum_{i=1}^{n} \widehat{\varepsilon}_{i}\widehat{\zeta}_{i}\right)^{2} \right)^{1/2}}\label{eq:T-pcm} \end{align*}

and reject the null by comparing to a standard normal distribution. In fact, as the target of $T$ in (3) is positive under conditional dependence, we perform a one-sided test which rejects when $T$ is large. The test statistic in (5) is approximately standard Gaussian if the regression methods employed for the $\widehat{f}(X, Z)$ on $Z$ and $Y$ on $Z$ are sufficiently predictive, the estimates $\widehat{f}$ are not too complicated and $Y \perp \hspace{-5pt}\perp X \,|\, Z$ [14, Theorem 4]. The test is powerful against alternatives where $\widehat{f}$ is correlated with the true $f$ and the aforementioned regression methods remain powerful [14, Theorem 5]. We summarize the procedure in Algorithm 2 below. (In this description and in Algorithm 2, we have omitted a few minor corrections to the estimation of $\widehat{f}$ that are done for numerical stability or as finite sample corrections. The full version of the algorithm with these additions is given in [14, Algorithm 1].) 


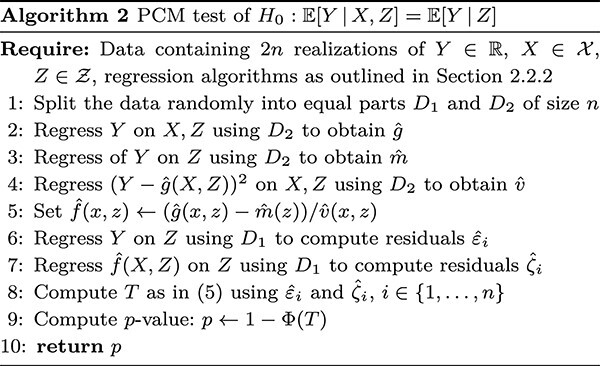


Due to the sample splitting, the $P$-value of the PCM is a random quantity. We can compute the PCM on several different splits to produce multiple $P$-values that can be dealt with using standard corrections for multiple testing. In practice, we follow the recommendation of the original paper and compute the $P$-value as in step 9 of Algorithm 2 but instead using the average of the test statistics from the different splits. We denote the number of different splits by $K$ and use 5–10 in the applications. The resulting test should be conservative that results in a power loss; however, the test averaged from different splits should still be more powerful than a single application of the PCM due to more efficient use of the data. If one desires a perfectly calibrated $P$-value from multiple splits, it is possible to use the method in [18] but we do not pursue this further here.

#### Comparison of the GCM and PCM tests

The GCM and PCM tests not only differ in terms of their target quantities, but also regarding computational aspects. The GCM test requires the regression of $Y$ on $Z$ and $X$ on $Z$. This prohibits the use of the GCM in settings where $X$ is a high-dimensional or non-tabular data modality and can not be represented as or reduced to a low-dimensional tabular modality. The PCM test, on the other hand, does not require regressing $X$ on $Z$. Thus, the PCM test allows the end-to-end use of non-tabular data modalities, such as images or text, for instance, via the use of deep neural networks. In contrast to the GCM, the PCM relies on sample splitting and requires more regressions and may thus be less data-efficient. This is addressed, in parts, by repeating the PCM test with multiple random splits, as described above.

### Data sets

#### Variable significance testing: CCLE data

We consider a subset of the anti-cancer drug dataset from the Cancer Cell Line Encyclopedia [CCLE, 19] that contains the response to the PLX4720 drug as a one-dimensional, continuous summary measure obtained from a dose-response curve and a set of $1638$ mutations (absence/presence coded as 0/1, respectively) in $n = 472$ cancer cell lines. To obtain comparable results, we follow the pre-processing steps in [20] and [21] by screening for mutations that are marginally correlated with drug response $S := \{j \in [1638]: \lvert \operatorname{Cor}(Y, X_{j})\rvert> 0.05\}$, which leaves $\lvert S\rvert = 466$ mutations. See Variable significance testing for a discussion of data-driven pre-screening of mutations on type-I error control.

#### Modality selection: TCGA data

We consider the openly available TCGA HCC multiomics data set used in [22, 23]. The preprocessed data consist of survival times for $n = 360$ patients with liver cancer together with RNA-seq ($\operatorname{RNAseq} \in{\mathbb{R}}^{15629}$), miRNA ($\operatorname{miRNA} \in{\mathbb{R}}^{365}$), and DNA methylation ($\operatorname{DNAm} \in{\mathbb{R}}^{19883}$) modalities. Pre-processing involved the removal of features and samples that contained more than 20% missing values and imputation of the remaining missing values. Further detail can be found in [22].

#### Modality selection with imaging: MIMIC data

We consider the MIMIC Chest X-Ray data set [24, 25], which contains the race ($R$; with levels ‘white’, ‘black’, ‘asian’), sex ($X_{1}$; with levels ‘male’, ‘female’), age ($X_{2}$, in years), pre-trained embeddings of chest X-rays ($Z$) and (among other response variables) whether a pleural effusion ($Y$) was visible on the X-ray for $n = 181\,342$ patients. The dimension of the image embedding was reduced by using the first 111 components of a singular value decomposition, which explain 98% of the variance.

### Computational details

All analyses were carried out using the R language for statistical computing [26]. The COMETs are implemented in comets [27], which relies on ranger [28] and glmnet [29] for the random forest (RF) and LASSO regressions, respectively. Code for reproducing all results is available at https://github.com/LucasKook/comets. In the following, unless specified otherwise, GCM and PCM tests are run with RFs for all regressions. LASSO regressions are used for analyzing the TCGA data in Modality selection. A Python implementation of COMETs, the pycomets library [30], is available on GitHub https://github.com/shimenghuang/pycomets.

## 3 Results

With our analyses, we aim to show how testing with covariance measures can be used to tackle two of the most common supervised learning problems in biomedical applications with multimodal data: Variable significance testing and modality selection (see Fig. 1). Throughout, we compare COMETs with existing methods (if applicable) on openly available real data sets (see Data sets for an overview of the data sets).

### Variable significance testing

We apply COMETs to the anti-cancer drug dataset from the Cancer Cell Line Encyclopedia [19] and compare with the results obtained using the CRT [10] GCIT [20], and DGCIT [21]. See Introduction for information on the CRT and Model-X based tests. The null hypotheses $H_{0}(j): Y \perp \hspace{-5pt}\perp X_{j} \,|\, X_{-j}$ are tested for $j \in S$ to detect mutations that are significantly associated with PLX4720 drug response.

#### COMETs identify mutations associated with PLX4720 drug activity

Table 1 summarizes the results for the GCIT, DGCIT, GCM, and PCM test and the 10 selected mutations in ([20], Fig. 4). Overall, there is large agreement between all tests that all reject the null hypothesis for the BRAF_V600E, BRAF_MC, HIP1, FLT3, THBS3, and DNMT1 mutations, corroborating previously reported results. For the PRKD1, PIP5K1A, and MAP3K5 mutations, the PCM test rejects, while the GCM test does not, which is consistent with the PCM test having power against a larger class of alternatives (Fig. 2).

**Table 1:** Results for the CCLE data in Variable significance testing. The table shows variable importance ranks and $P$-values for the relation of mutations of 10 genes with the response to PLX4720 conditional on the 465 other mutations in the data. The PCM test was run with $K = 10$ random splits. The variable importance ranks (obtained via random forests, RF, or elastic net regression, EN) and the CRT, GCIT, and DGCIT results were obtained from [20] and [21].

Method	Gene mutations									
	BRAF_V600E	BRAF_MC	HIP1	FLT3	CDC42BPA	THBS3	DNMT1	PRKD1	PIP5K1A	MAP3K5
EN	1	3	4	5	7	8	9	10	19	78
RF	1	2	3	14	8	34	28	18	7	9
CRT	$<0.001$	$<0.001$	$0.008$	0.017	0.009	0.017	0.022	0.002	0.024	0.012
GCIT	$<0.001$	$<0.001$	$0.008$	0.521	0.050	0.013	0.020	0.002	0.001	$< 0.001$
DGCIT	$<0.001$	$<0.001$	$<0.001$	$<0.001$	$<0.001$	$<0.001$	$<0.001$	$<0.001$	$<0.001$	0.794
GCM	0.030	0.033	0.010	0.005	0.004	0.042	0.010	0.165	0.464	0.504
PCM	0.001	0.012	0.008	0.009	0.014	0.027	0.014	0.011	0.022	0.022
GCM (no screening)	0.003	0.021	0.006	0.002	0.002	0.068	0.007	0.007	0.223	0.216
PCM (no screening)	0.002	0.007	0.082	0.151	0.186	0.134	0.138	0.108	0.198	0.122

#### COMETs detect relevant mutations without pre-screening

Prior results rely on pre-screening genes based on their marginal correlation with the drug response. However, marginal correlation cannot inform subsequent conditional independence tests in general and the data-driven pre-screening may have lead to inflated false positive rates [31]. However, the GCM and PCM test can be applied without pre-screening and still consistently reject the null hypothesis of conditional independence for the BRAF_V600E and BRAF_MC mutations (see rows in Table 1 with ‘no screening’). When correcting (Holm) the $P$-values to attain a family-wise error rate of $5\%$ for the 10 mutations of interest, the GCM and PCM still reject the null hypothesis for BRAF_V600E ($p = 0.024$ for the GCM test and $p = 0.020$ for the PCM test). This rejection is expected because PLX4720 was designed as a BRAF inhibitor [19].

### Modality selection

The goal of our analysis is to identify modalities among RNA-seq, miRNA and DNA methylation that are important for predicting survival of liver cancer patients by testing if the event is independent of the modality $M_{j}$, given the other modalities $M_{-j}$, $j \in \{1, 2, 3\}$. This is a challenging problem due to the high dimensionality of both the candidate modality $M_{j}$ and the conditioning variables in $M_{-j}$.

#### Evidence of DNA methylation being important for predicting survival in liver cancer patients

Table 2 (PCM-RF) shows $P$-values for the PCM test ($K=10$ different splits) testing for significance of the RNA-seq, miRNA, and DNA methylation modalities conditional on the remaining two without pre-screening features in any of the modalities using a RF regression. There is some evidence that the DNA methylation modality is important for predicting death in liver cancer patients. Conversely, the PCM test does not provide evidence that survival depends on the RNA-seq or miRNA modalities, when already conditioning on the DNA methylation data. Comparable results are obtained when substituting the RF regression for $Y$ on $\operatorname{RNAseq}$, $\operatorname{miRNA}$, and $\operatorname{DNAm}$ with a cross-validated LASSO regression using the optimal tuning parameter: after a multiple testing correction (Holm), both PCM tests reject the null hypothesis only for the DNA methylation modality.

**Table 2:** Results ($P$-values) for the multiomics application in Modality selection using the PCM with $K=10$ random splits once using an RF for the regression of $Y$ on $\operatorname{RNAseq}$, $\operatorname{miRNA}$ and $\operatorname{DNAm}$, and once a cross-validated high-dimensional linear regression (LASSO).

Null hypothesis	PCM-RF	PCM-LASSO
$H_{0}: Y \perp \hspace{-5pt}\perp \operatorname{RNAseq} \,|\, \operatorname{miRNA}, \operatorname{DNAm}$	0.178	0.066
$H_{0}: Y \perp \hspace{-5pt}\perp \operatorname{miRNA} \,|\, \operatorname{RNAseq}, \operatorname{DNAm}$	0.165	0.044
$H_{0}: Y \perp \hspace{-5pt}\perp \operatorname{DNAm} \,|\, \operatorname{RNAseq}, \operatorname{miRNA}$	0.014	0.002

### Modality selection with imaging data

Using deep learning methods, [32] provide evidence that both the race and the response (pleural effusion) can be predicted from the X-ray embedding with high accuracy. The goal of our analysis is to test, whether race helps predict the response when already conditioning on age, sex, and the X-rays and, vice versa, whether the X-rays contain information for predicting pleural effusion given sex, age, and race.

#### Strong evidence for X-ray imaging and race being important for predicting pleural effusion

There is strong evidence against the null hypotheses of pleural effusion being independent of either X-ray imaging or race, given the other and, additionally, sex and age of a patient (Table 3).

**Table 3:** Results ($-\log _{10}$-transformed $P$-values) for the GCM and PCM applied to the full MIMIC data set in Modality selection with imaging data. Both tests reject both hypotheses. See Fig. 3 for an uncertainty assessment.

Null hypothesis	GCM	PCM
$H_{0}: Y \perp \hspace{-5pt}\perp R \,|\, X, Z$	6.158	77.762
$H_{0}: Y \perp \hspace{-5pt}\perp Z \,|\, X, R$	13805.802	1270.361

To gauge the uncertainty in the results of the COMETs, we repeat the tests on 75 random (non-overlapping) subsamples of different sample sizes (150, 600, 2400) of the data. Only the PCM rejects the null hypothesis of pleural effusion (PE) being independent of race given the X-ray, sex, and age of a patient at any of the considered sample sizes, which provides evidence that ${\mathbb{E}}[\varepsilon \xi ]$ is close to zero yet ${\mathbb{E}}[Y \,|\, X,Z]$ still varies non-linearly with $X$. At full sample size, the GCM does reject, indicating the presence of a weak linear signal (estimated correlations between pleural effusion and race residuals are smaller than $0.015$). It is somewhat unsurprising to see both COMETs reject the null hypothesis at such large sample sizes ([33], Modality selection with imaging data).

**Figure 3:**
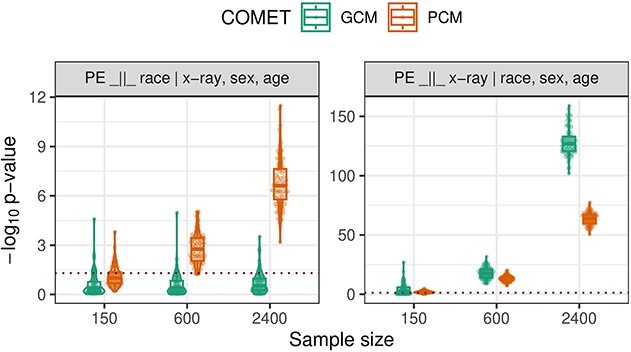
Results ($-\log _{10}$-transformed $P$-values) for the GCM and PCM applied to 75 random non-overlapping splits of different sample sizes ($n \in \{150, 600, 2400\}$) of the MIMIC data set in Modality selection with imaging data. Splitting the data enables an analysis of the uncertainty in the tests’ rejections and the strength of evidence against the null.

Both tests reject the null hypothesis of pleural effusion (PE) being independent of X-ray given race, sex and age of a patient at any sample size but in fact the GCM produces smaller $P$-values. This indicates that there is a significant component in ${\mathbb{E}}[Y \,|\, X, Z]$ varying linearly with $X$; in these cases, the PCM will not outperform the GCM for a fixed sample size.

### Computation times

**Figure 4:**
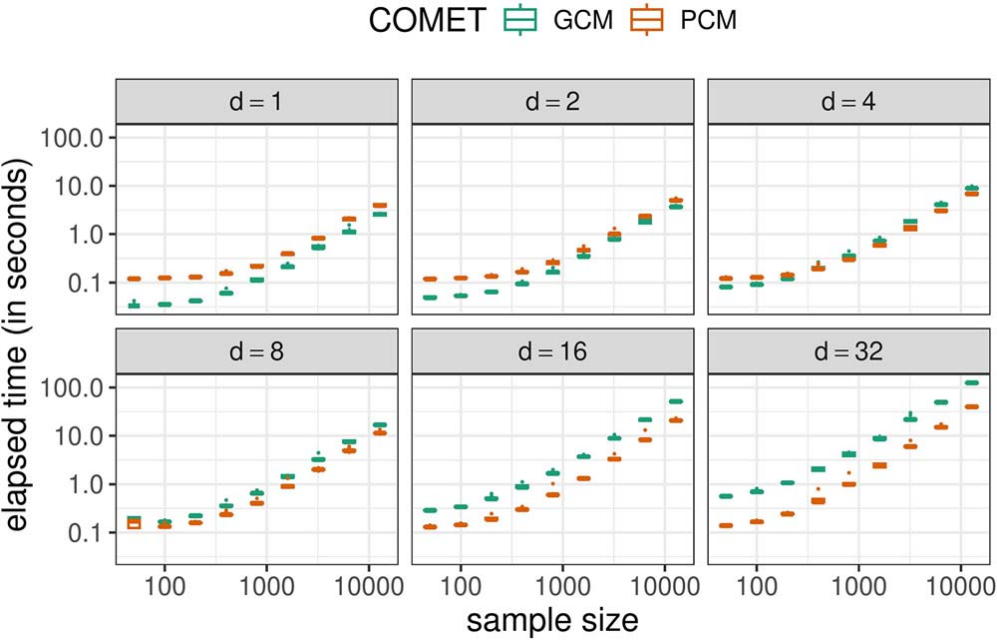
Computation times (in seconds; y-axis) for the GCM and PCM test using random forest regressions for varying dimensionality of $X$ (panels) and sample size (x-axis).

The computation time of the GCM and PCM test depends on the dimensionality $d$ of $X \in{\mathbb{R}}^{d}$ and sample size $n$ and the chosen regression methods. For low-dimensional $X$, the PCM test requires more regressions than the GCM test which results in slower computation times (see Fig. 4). However, for higher-dimensional $X$, the GCM test requires more regressions resulting in longer computation times. For moderate dimensions ($d = 4$ and $8$), the computation times are similar.

## Discussion

We present COMETs for algorithm-agnostic significance testing with multi-modal, potentially non-tabular data, which relies on tests of conditional independence based on covariance measures. The versatility of the GCM and PCM tests is shown in several applications involving variable significance testing and modality selection in the presence of high-dimensional conditioning variables. In the following, we discuss the applications in more detail and end with a discussion of computational aspects and recommendations for using COMETs in supervised learning applications with multimodal data.

### Variable significance testing

The GCM and PCM test show comparable results to competing methods and can be applied without relying on data-driven pre-screening which, otherwise, can invalidate $P$-values and lead to inflated type I error rates. Type I error control additionally suffers from the performed number of tests. After correcting for multiple testing, the COMETs provide evidence that BRAF_V600E is associated with PLX4720 activity while controlling for all other mutations. As highlighted before, this is expected since PLX4720 was designed as a BRAF inhibitor.

### Modality selection

The PCM test is applied to the TCGA data set to test which modalities (RNAseq, miRNA, DNAm) are important (conditional on the others) for predicting survival in liver cancer patients and rejects the null hypothesis for the DNA methylation modality. Failure to reject the null hypothesis for the RNA-seq and miRNA modalities may be due to the low sample size and extremely high dimensionality of the problem and ought to be interpreted as lack of evidence that RNA-seq and miRNA data contain information for predicting survival beyond DNA methylation in the data at hand. Taken together, this application demonstrates that the PCM test can be used for modality selection with high-dimensional candidate and conditioning modalities. COMETs could, for instance, be used to trade off the economic cost of measuring an omics (or imaging, as in the MIMIC application) modality with the gain in predictive power at a given significance level. It is worth noting that a naive test based on the comparison of cross-validated mean-squared errors using all variables and all but one variable does not result in a valid statistical test [12, 14]. Lastly, the validity of conditional independence tests applied to the TCGA data depends on the validity of the imputation procedure used during data pre-processing.

### Modality selection with imaging data

The large and openly available MIMIC data set serves as an example application of how image and other non-tabular modalities may enter an analysis based on COMETs. The PCM does not require pre-trained embeddings and could, in principle, also be used in combination with deep convolutional neural networks if the raw imaging data is available. The 111-dimensional embedding further enables the use of the GCM test to serve as a benchmark. However, it is important to properly choose the regressions involved in COMETs as the tests rely on their quality and asymptotic properties [14, 15]. Nevertheless, to the best of our knowledge, no other tests exist with theoretical guarantees that also permit testing $Y \perp \hspace{-5pt}\perp X \,|\, Z$ when $X$ is a non-tabular modality.

### Recommendations and outlook

As outlined in Comparison of the GCM and PCM tests, the regression of $X$ on $Z$ required by the GCM can become computationally challenging if $X$ is high-dimensional (which is why the GCM test is not applied in Modality selection for modality selection) or non-tabular (this was circumvented by using the relatively low-dimensional tabular embedding of the chest X-ray images in Modality selection with imaging data; see also the computation times in Computation times). The PCM test, in contrast, does not rely on this regression and is thus directly applicable in cases where $X$ and $Z$ are high-dimensional or non-tabular modalities. The GCM has further been adapted to settings with functional outcomes [34], continuous time stochastic processes [35], censored outcomes [36], and extended to powerful weighted [16] and kernel-based [37] versions. These are all COMETs proposed in the literature and we leave their applicability in biomedical contexts as a topic for future work.

In the applications presented in this paper, RF and LASSO regressions were used. RFs are computationally fast and require little hyperparameter tuning to obtain well-performing regression estimates. However, for very high-dimensional applications in which the number of features exceeds the number of observations, the LASSO is a fast and computationally stable alternative.

Overall, we believe that COMETs provide a useful tool for bioinformaticians to assess significance in applications with high-dimensional and potentially non-tabular omics and biomedical data while appropriately controlling error probabilities. The increasing familiarity of data analysts with supervised learning methods, on which COMETs rely, help safeguard the validity of the statistical inference. Further, the algorithm-agnostic nature of the procedures makes COMETs easily adaptable to future developments in predictive modeling.

**Key Points**

- We show how COvariance MEasure Tests (COMETs) for conditional independence can be applied for the ubiquitous tasks of variable significance testing and modality selection in high-dimensional multimodal and non-tabular datasets.
- The algorithm-agnostic nature of the COMETs allow the data analyst to control for complex high-dimensional confounders with potentially non-linear confounding mechanisms.
- Using COMETs, we (i) screen for the significance of mutations in predicting PLX4720 drug activity in the CCLE dataset, (ii) select entire omics modalities for predicting survival in liver cancer patients in the TCGA dataset, and (iii) investigate the significance of image and tabular modalities for predicting the presence of pleural effusion in the MIMIC dataset.
- We provide a user-friendly open source implementation of several covariance measure tests in both R and Python to foster their use and usability in the bioinformatics community. We give recommondations for choosing and tuning the supervised learning algorithms used in COMETs.
